# Supplementary material for: Splice-Junction-Based Mapping of Alternative Isoforms in the Human Proteome
Source: Cell Rep. Author manuscript; Available in PMC 2020 Jan 15. (PMC6961840; doi:10.1016/j.celrep.2019.11.026)
Supplement: 2 [file NIHMS1546469-supplement-2.zip › DF1/PXD006675/PulmonaryVein/PulmonaryVein_11_SYPL1_ETSLHSPSNTSAPHSQGGIPPPTGL.pdf]

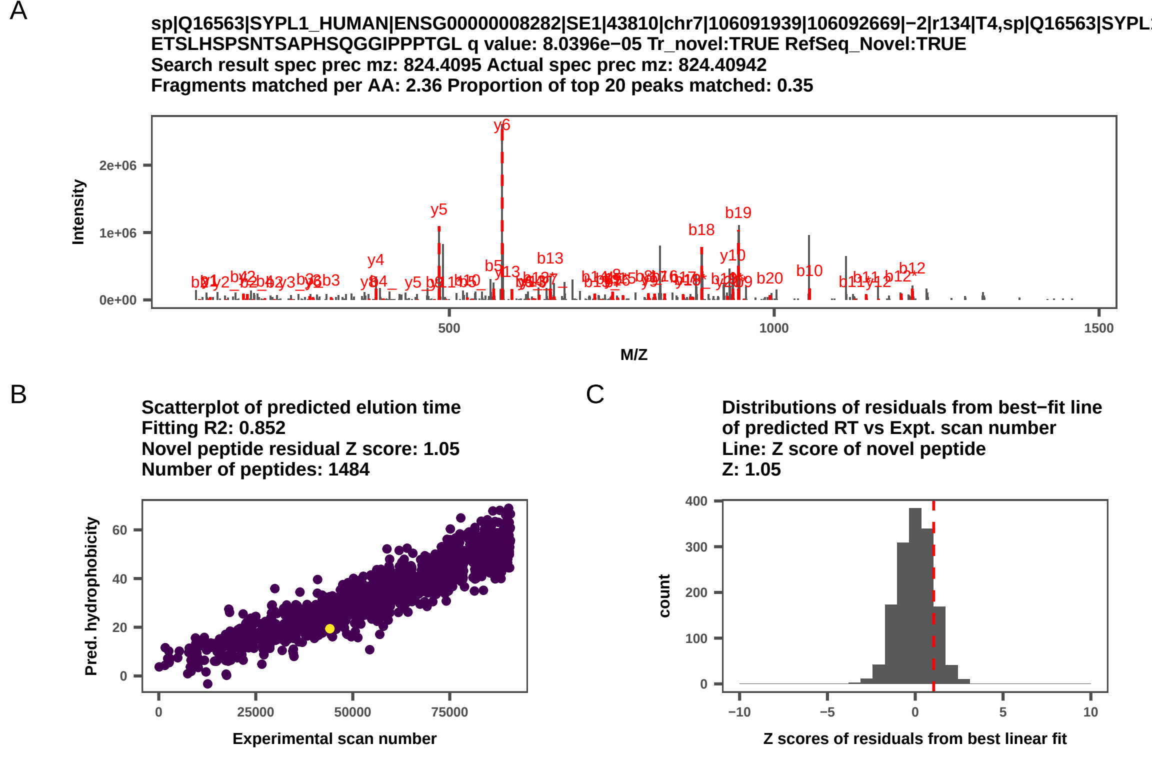
A
sp|Q16563|SYPL1_HUMAN|ENSG00000008282|SE1|43810|chr7|106091939|106092669|−2|r134|T4,sp|Q16563|SYPL
ETSLHSPSNTSAPHSQGGIPPPTGL q value: 8.0396e−05 Tr_novel:TRUE RefSeq_Novel:TRUE
Search result spec prec mz: 824.4095 Actual spec prec mz: 824.40942
Fragments matched per AA: 2.36 Proportion of top 20 peaks matched: 0.35
Intensity
2e+06
1e+06
0e+00
M/Z
500
1000
1500
y6
y5
b19
b18
y10
b13
b10
b12
b20
B
Scatterplot of predicted elution time
Fitting R2: 0.852
Novel peptide residual Z score: 1.05
Number of peptides: 1484
Pred. hydrophobicity
Experimental scan number
0
25000
50000
75000
20
40
60
C
Distributions of residuals from best−fit line
of predicted RT vs Expt. scan number
Line: Z score of novel peptide
Z: 1.05
count
400
300
200
100
0
Z scores of residuals from best linear fit
−10
−5
5
10
